# Human and Viral Golgi Anti-apoptotic Proteins (GAAPs) Oligomerize via Different Mechanisms and Monomeric GAAP Inhibits Apoptosis and Modulates Calcium*[Fn FN2]

**DOI:** 10.1074/jbc.M112.414367

**Published:** 2013-03-18

**Authors:** Nuno Saraiva, David L. Prole, Guia Carrara, Carlos Maluquer de Motes, Benjamin F. Johnson, Bernadette Byrne, Colin W. Taylor, Geoffrey L. Smith

**Affiliations:** From the ‡ Department of Pathology, University of Cambridge, Tennis Court Road, Cambridge CB2 1QP, United Kingdom,; the § Section of Virology, Department of Medicine, Imperial College London, Norfolk Place, London W2 1PG, United Kingdom,; the ¶ Department of Pharmacology, University of Cambridge, Tennis Court Road, Cambridge CB2 1PD, United Kingdom, and; the ‖ Division of Molecular Biosciences, Imperial College London, London SW7 2AZ, United Kingdom

**Keywords:** Apoptosis, Calcium, Golgi, Protein Complexes, Viral Protein, Bax Inhibitor 1, Golgi Anti-apoptotic Protein, Oligomerization, Poxvirus, TMBIM

## Abstract

Golgi anti-apoptotic proteins (GAAPs) are hydrophobic proteins resident in membranes of the Golgi complex. They protect cells from a range of apoptotic stimuli, reduce the Ca^2+^ content of intracellular stores, and regulate Ca^2+^ fluxes. GAAP was discovered in camelpox virus, but it is highly conserved throughout evolution and encoded by all eukaryote genomes examined. GAAPs are part of the transmembrane Bax inhibitor-containing motif (TMBIM) family that also includes other anti-apoptotic and Ca^2+^-modulating membrane proteins. Most TMBIM members show multiple bands when analyzed by SDS-PAGE, suggesting that they may be oligomeric. However, the molecular mechanisms of oligomerization, the native state of GAAPs in living cells and the functional significance of oligomerization have not been addressed. TMBIM members are thought to have evolved from an ancestral GAAP. Two different GAAPs, human (h) and viral (v)GAAP were therefore selected as models to examine oligomerization of TMBIM family members. We show that both hGAAP and vGAAP in their native states form oligomers and that oligomerization is pH-dependent. Surprisingly, hGAAP and vGAAP do not share the same oligomerization mechanism. Oligomerization of hGAAP is independent of cysteines, but oligomerization of vGAAP depends on cysteines 9 and 60. A mutant vGAAP that is unable to oligomerize revealed that monomeric vGAAP retains both its anti-apoptotic function and its effect on intracellular Ca^2+^ stores. In conclusion, GAAP can oligomerize in a pH-regulated manner, and monomeric GAAP is functional.

## Teaser

**Background:** GAAPs are Golgi anti-apoptotic proteins that modulate Ca^2+^ signaling.

**Results:** Unlike human GAAP, viral GAAP oligomerization is cysteine-dependent. Monomeric vGAAP is functional.

**Conclusion:** Viral and human GAAPs show pH-dependent oligomerization via different mechanisms. Monomeric vGAAP is anti-apoptotic and modulates intracellular Ca^2+^.

**Significance:** This is the first mechanistic analysis of TMBIM protein oligomerization, and shows that monomers are functional.

## Introduction

The gene encoding Golgi anti-apoptotic protein (GAAP)^4^ was initially identified in camelpox virus (CMLV) and some vaccinia virus (VACV) strains (1). Deletion of the *GAAP* gene from the VACV genome had no effect on virus replication in cell culture but altered virus virulence *in vivo* (1). Cells expressing viral GAAP (vGAAP) displayed resistance to apoptosis triggered by a wide range of intrinsic and extrinsic stimuli (1).

These viral proteins are closely related (73% amino acid identity) to the ubiquitously expressed human GAAP (hGAAP) (1). hGAAP was identified as a possible housekeeping gene because it is essential for cell viability (1) and from statistical analysis of microarrays (2). Furthermore, its mRNA levels are dysregulated in some human breast tumors, making it a putative oncogene and a possible target for anti-cancer therapy (3).

GAAPs are highly conserved, and closely related proteins are predicted to be encoded by the genomes of fungi, plants, and animals (1). All GAAPs, from evolutionarily diverse sources, have similar lengths and hydrophobicity profiles that point to important and conserved functions. Cells in which hGAAP was knocked down using siRNA died but were rescued by expression of vGAAP (1), suggesting that the two GAAPs are functionally interchangeable.

Phylogenetic analysis suggests that GAAPs are ancient within eukaryotes. Some members of the transmembrane Bax (Bcl-2-associated X protein) inhibitor-containing motif (TMBIM) family may have derived from a GAAP ancestor ∼2,000 million years ago (4). Members of the TMBIM family include TMBIM1 or RESC (responsive to centrifugal force and shear stress gene 1 protein), TMBIM2 or LFG (lifeguard protein), TMBIM3 or GRINA (glutamate receptor ionotropic NMDA-associated protein), TMBIM4 or GAAP, TMBIM5 or GHITM (growth hormone-inducible transmembrane protein), and TMBIM6 or BI-1 (Bax inhibitor 1) (1, 4–7). All TMBIM proteins are predicted to have similar secondary structures, consisting of multiple transmembrane domains (TMDs) with short interconnecting loops, a charged C terminus, and a conserved UPF0005 motif with unknown function that stretches from the beginning of the third to the middle of the fourth TMD (4, 8). Recently, vGAAP, hGAAP, and BI-1 were shown to have a six TMD topology with cytosolic N and C termini and a likely re-entrant loop at the C terminus (9).

Similar to GAAP, BI-1 is a widely expressed and evolutionarily conserved protein that protects cells from many different intrinsic and extrinsic death stimuli (5). It is the most studied member of the TMBIM family. BI-1 localizes primarily to endoplasmic reticulum (ER) membranes, where it causes pH-dependent Ca^2+^ leakage that reduces the ER Ca^2+^ content, and so the amount of Ca^2+^ released upon stimulation (10). Work with purified BI-1 reconstituted into liposomes suggests it has a Ca^2+^/H^+^ antiporter-like (11) or Ca^2+^ channel activity (12). Moreover, two aspartic acid residues (Asp^209^ and Asp^213^) in BI-1 are essential for Ca^2+^ flux from the ER (12). Regulation of the Ca^2+^ content of the ER by BI-1 suggests a possible mechanism for its anti-apoptotic function.

GAAPs localize primarily to Golgi membranes and, similar to BI-1, have a broad spectrum of anti-apoptotic activity. This suggests a possible link with intracellular Ca^2+^, which plays important roles in apoptosis triggered by different stimuli (13). This suggestion is also consistent with measurements of the Ca^2+^ content of organelles after overexpression or knockdown of hGAAP (14). Overexpression of hGAAP reduced the Ca^2+^ content of the Golgi and ER and the amount of Ca^2+^ released by an apoptotic stimulus (14). Therefore, the effects of GAAPs on Ca^2+^ signaling might explain their broad anti-apoptotic activity, although a causal link is yet to be established.

The membrane topology of TMBIM proteins has been established (9), but there are no further structural data. Most TMBIM proteins show multiple bands on SDS-PAGE, suggesting the existence of oligomers. However, neither the oligomeric state of TMBIM proteins in native membranes nor the determinants or functional consequences of oligomerization are known. To address these questions, the mechanisms of oligomerization of vGAAP and hGAAP were studied in biochemical and cell-based assays. Data presented reveal that both vGAAP and hGAAP oligomerized in a pH-dependent manner. However, the mechanisms of oligomerization are different for vGAAP and hGAAP. Disulfide bonds, and specifically cysteine residues 9 and 60, are required for vGAAP but not for hGAAP oligomerization. In functional assays, a mutant vGAAP in which oligomerization was prevented retained both its anti-apoptotic activity and its ability to modulate the Ca^2+^ content of intracellular stores. We conclude that monomeric GAAP can regulate both Ca^2+^ signaling and apoptosis.

## EXPERIMENTAL PROCEDURES

### 

#### 

##### Plasmids

The pcDNA3.1 plasmids containing vGAAP-HA and hGAAP-HA were described previously (1). Plasmids encoding C-terminal HA fusions of GAAP TMD1–4 (vGAAP residues 1–146 and hGAAP residues 1–145) and TMD5–6 (vGAAP residues 147–237 and hGAAP residues 145–238) were generated by inserting PCR products of truncated vGAAP and hGAAP into the BamHI/EcoRI restriction sites of pcDNA3.1. Site-directed mutagenesis was performed using the QuikChange multi site-directed mutagenesis kit (Stratagene). PCR products of wild type and mutant vGAAP and hGAAP were subcloned into the NheI/AgeI restriction sites of pEGFP-C1 and ptdTomato-N1 (Clontech) to obtain GAAPs C-terminally tagged with GFP or tandem Tomato (Tom). These plasmids encode a flexible linker (GGSGGSGGSKR) between the GAAP and the fluorescent tag to allow folding flexibility. The coding sequences of all plasmids were confirmed by sequencing. A plasmid encoding GRASP65-GFP was a kind gift from Dr. Theresa Ward (London School of Hygiene and Tropical Medicine).

##### Cell Culture and Transfections

HeLa and U2-OS cells were maintained in MEM and DMEM, respectively, supplemented with 10% heat-treated (56 °C, 1 h) FBS and 1% penicillin-streptomycin (Invitrogen). HeLa cells were also supplemented with 2 mm l-glutamine. Plasmid transfections were carried out using FuGENE6 (Roche Diagnostics) according to the manufacturer's instructions.

##### Stable Cell Lines

HeLa polyclonal stable cell lines expressing C-terminally V5-tagged vGAAP or hGAAP were generated using the HIV-1-based lentiviral vector pdlNot′MCS′R′PK as described (9, 15). The HeLa A36-V5 cell line was described previously (15). The U2-OS stable cell lines expressing wild type or mutant vGAAP that were used in apoptosis assays were generated using a lentiviral bicistronic vector coding for GFP in the second cistron (a gift from Dr. Heike Laman, University of Cambridge). Transduced cells were sorted based on GFP expression using a MoFlo MLS high-speed cell sorter (Beckman Coulter) to obtain a population in which >95% of cells expressed GFP.

##### Protein Purification

CMLV GAAP was expressed in *Saccharomyces cerevisiae* cultures engineered to express a cleavable C-terminal GFP-His_8_-tagged vGAAP under control of the galactose promoter, as described for other transmembrane proteins (16). The protein was extracted in lauryldimethylamine oxide, purified, and analyzed according to the protocol developed by Drew *et al.* (16). The GFP-His_8_ tag was removed by adding His_8_-tagged tobacco etch virus protease to the purified vGAAP-His_8_-GFP at a molar ratio of 1:1 of GFP and tobacco etch virus protease and digested overnight at 4 °C. The presence of GFP-tagged proteins in protein samples resolved on 12% polyacrylamide Tris-glycine gels (Invitrogen) was assessed by in-gel GFP fluorescence. The gel was exposed to blue light (460 nm, cut-off filter 515 nm) for 10–60 s, and the image was captured with a LAS-1000–3000 charge-coupled device imaging system (Fujifilm).

##### Immunoblots

Cells were lysed on ice in lysis buffer comprising 50 mm Tris-HCl, pH 7.5, 100 mm NaCl, 2 mm EDTA, 1% CHAPS (w/v), protease and phosphatase inhibitor mixtures (Roche). The lysates were cleared by centrifugation at 15,000 × *g* for 15 min, mixed with 5× reducing (0.01–5 mm DTT) or non-reducing (no DTT) loading buffer, resolved by SDS-PAGE, and transferred onto a nitrocellulose membrane. Antigen-antibody complexes were detected using RDye-conjugated secondary antibodies and a Li-COR scanner (Odyssey). The following antibodies and dilutions were used: 1:5000 anti-HA (Sigma), 1:5000 anti-V5 (AbD Serotec, Ltd.), 1:10,000 anti-tubulin (Millipore), 1:1000 anti-GAAP (1), 1:2500 anti-GFP (Clontech), and 1:2000 anti-FLAG (Sigma).

##### Co-immunoprecipitation

HeLa cells stably expressing V5-tagged hGAAP or vGAAP were transfected with the appropriate plasmids, and 18 h later were lysed in immunoprecipitation (IP) buffer (50 mm Tris-HCl, pH 7.5, 500 mm NaCl, 2 mm EDTA, 1% CHAPS (w/v), protease and phosphatase inhibitor mixtures (Roche Diagnostics)). The total protein content was normalized after measurements with a BCA assay (Pierce). The anti-V5 antibody was added to the lysates and incubated overnight. Protein G-Sepharose beads (Roche Diagnostics) were incubated with the lysates for 2 h at 4 °C, washed four times with 1 ml of IP buffer, resuspended in 5× reducing loading buffer, and resolved by SDS-PAGE.

##### Protein Cross-linking

HeLa cells transfected with pcDNA3.1 encoding vGAAP-HA or hGAAP-HA were collected after 18 h, lysed in lysis buffer, and incubated with the lysine-reactive aryl halide 1,5-difluoro-2,4-dinitrobenzene (DFDNB, 2.5–20 nm, 20 min at 37 °C). The reaction was stopped by addition of 20 mm Tris, pH 7.5.

##### Förster Resonance Energy Transfer

For FRET, near-confluent cultures of HeLa cells were transfected using FuGENE 6 (Roche Applied Science) on glass coverslips (VWR) with a pair of plasmids encoding GFP- (0.5 μg) or Tom-tagged proteins (1.5 μg). These fluorescent proteins are a FRET pair, with GFP as donor and Tom as acceptor fluorophore (17). After 18 h, cells were fixed in 4% paraformaldehyde and imaged using an Olympus IX81 microscope with a 60 × 1.45 numerical aperture objective and illumination with 488-nm or 561-nm diode-based lasers via appropriate filters. Images were acquired with an Andor iXon 897 camera and processed using Cell'R software (Olympus, Milton Keynes, UK). To determine FRET efficiency, images of each cell were acquired using 561 and 488 nm excitation, before and after photobleach of Tom by exposure to the unattenuated 561-nm laser for 60 s. At least 10 cells were imaged for each condition, and for each cell, a region corresponding to the Golgi was selected for analysis. FRET efficiency (*E*) was determined using the following formula,

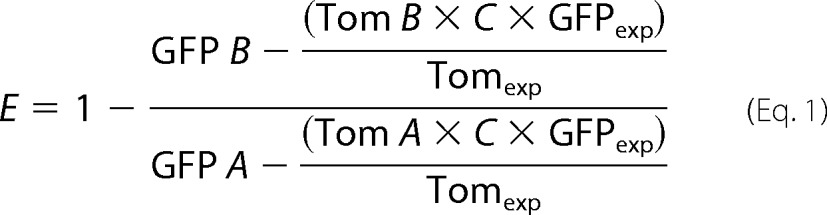
 where *B* denotes fluorescence intensity before photobleach; *A* denotes fluorescence intensity after photobleach; exp denotes units of exposure, and *C* denotes the fraction of Tom excitation from the 488-nm laser, relative to the 561-nm laser, per unit of exposure. Gray value images were obtained from pixel-by-pixel subtraction of GFP pre-bleach from GFP post-bleach images using NIH ImageJ software.

##### Measurements of [Ca^2+^]_i_

For measurement of intracellular free Ca^2+^ concentration ([Ca^2+^]*_i_*) in cell populations, cells from a confluent 75-cm^2^ flask of HeLa cells were resuspended in 200 μl of Hank's balanced salt solution (Invitrogen) and transfected with 0.5 μg DNA/10 μl cells using Neon nucleofection (Invitrogen). Transfected cells (10 μl/well) were seeded into 96-well plates, and after 24 h, when almost confluent, they were incubated with 6 μm Fura-2/AM (Invitrogen) for 40 min at 37 °C. Cells were washed, and measurements of [Ca^2+^]*_i_* were performed at 20 °C in Ca^2+^-free medium comprising 132 mm NaCl, 5 mm KCl, 2 mm, MgCl_2_, 10 mm glucose, 10 mm HEPES, 1 mm EGTA, pH 7.2. Fluorescence (excitation at 340 nm and 380 nm; emission at 510 nm) was measured using a fluorescence plate reader (FlexStation 3, MDS Analytical Devices). Background fluorescence was determined by addition of ionomycin (1 μm) with MnCl_2_ (10 mm), and background-corrected fluorescence ratios (*F*_340_/*F*_380_) were calibrated to [Ca^2+^]*_i_* using Ca^2+^ standard solutions (Invitrogen) and a *K_D_* for Ca^2+^ of 236 nm (18).

##### Apoptosis Assays

U2-OS cells were seeded in 96-well plates (10^3^ cells/well). After 48 h, cells were treated with staurosporine (0.5 μm, 6–8 h), doxorubicin (3 μm, 48 h), or vehicle. Extrinsically triggered apoptosis was induced by incubation with cycloheximide (20 μg/ml) and TNFα (10 ng/ml) for 16 h. Caspase activity was then determined using Caspase-Glo 3/7 substrate (Promega).

##### Bioinformatics

Multiple sequence alignments were performed using ClustalW (version 1.83).

##### Immunofluorescence

Transfected cells were fixed with 4% paraformaldehyde and stained with 1:300 anti-GM130 (BD Biosciences) or 1:300 anti-HA (Cambridge Biosciences) and with secondary antibodies (1:500) conjugated to Alexa Fluor 488 or Alexa Fluor 568 (Invitrogen). The coverslips were mounted in Mowiol 4–88 containing DAPI to stain nuclei. Cells were imaged by confocal microscopy using an LSM 5 PASCAL microscope (Zeiss). Images were acquired using the LSM image browser software (Zeiss).

## RESULTS

### 

#### 

##### GAAPs Form Oligomers

**FIGURE 1.**
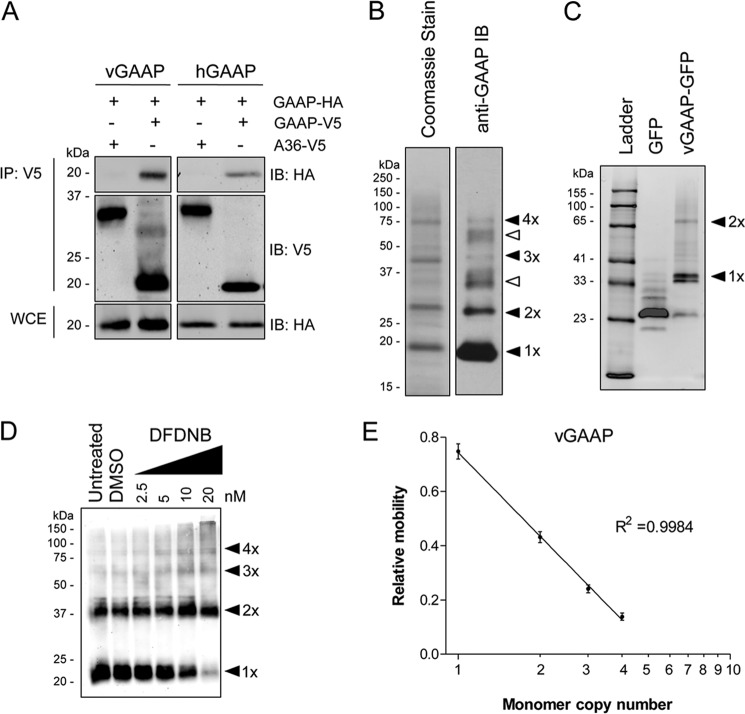
**GAAPs form oligomers.** *A*, HeLa cells stably expressing hGAAP-V5 or vGAAP-V5 were transfected with plasmids encoding hGAAP-HA or vGAAP-HA. After 16 h, cells were lysed and subjected to IP using anti-V5 antibody. A HeLa cell line expressing Ala^36^-Val^5^ (∼37 kDa), a VACV membrane protein, was used as a control for nonspecific IP of membrane proteins. Immunoblots (*IB*) used anti-HA or anti-V5 antibodies. Whole cell extract (*WCE*) was also immunoblotted with anti-HA. *B*, CMLV vGAAP was expressed and purified from yeast. The protein was resolved by non-reducing SDS-PAGE and analyzed by Coomassie staining and by immunoblotting with anti-GAAP antibody. *Filled arrowheads* show the expected positions of the indicated oligomeric forms of GAAP. *Open arrowheads* show positions of residual amounts of uncleaved GAAP-GFP in its monomeric and dimeric forms. *C*, the vGAAP-GFP fusion protein before tobacco etch virus protease cleavage was analyzed by in gel fluorescence. Results shown are representative of three independent experiments. *Arrowheads* show the expected positions of monomeric and dimeric GAAP-GFP. *D*, HeLa cells were transfected with a plasmid encoding vGAAP-HA and analyzed 18 h later. Cell lysates were incubated for 20 min with DFDNB, dimethyl sulfoxide (*DMSO*), or untreated, and subjected to non-reducing SDS-PAGE, prior to immunoblotting with anti-HA antibody. The predicted oligomeric states of GAAP are shown by *arrowheads. E*, the relative mobilities of the different oligomeric forms were determined and plotted against their predicted oligomeric states. Results are from three independent experiments and presented as means ± S.E. The sizes of molecular mass markers are shown in kDa.

To investigate whether GAAPs oligomerize, plasmids encoding HA-tagged vGAAP or hGAAP were transfected into HeLa cells stably expressing vGAAP or hGAAP with a V5 tag (GAAP-V5). Cells stably expressing A36, another VACV membrane protein (19), with a V5 tag (15), were used as controls for nonspecific pulldown of membrane proteins. Proteins were immunoprecipitated using anti-V5 antibody, and the immunoprecipitates were analyzed by SDS-PAGE and immunoblotting with anti-HA antibody. HA-tagged vGAAP and hGAAP co-immunoprecipitated with V5-tagged vGAAP and hGAAP, respectively, but not with V5-tagged A36 (Fig. 1*A*). These results indicate that vGAAP and hGAAP interact, directly or indirectly, with themselves.

To determine whether the association between GAAPs is direct, CMLV vGAAP was expressed in yeast, purified, and separated by non-reducing SDS-PAGE. Four bands were observed, with sizes that were consistent with oligomeric forms of the protein (Fig. 1*B*), and each band reacted with an anti-GAAP antibody. These results are consistent with the presence of monomeric, dimeric, trimeric, and tetrameric forms of GAAP (Fig. 1*B*), but they do not exclude heteromeric assembly of GAAP with another protein of similar size. However, the latter possibility is unlikely given the results from in-gel fluorescence of purified CMLV vGAAP-GFP (Fig. 1*C*). Under non-reducing conditions, these gels also revealed the presence of dimers of the larger GAAP-GFP proteins (Fig. 1*C*). Together, these results suggest that the interactions observed by co-IP are due to a direct interaction between GAAPs.

In an attempt to characterize the stoichiometry of GAAP oligomerization, the lysine-reactive aryl halide DFDNB cross-linker was used (Fig. 1*D*). Because this hydrophobic cross-linker has a very short spacer arm and infiltrates membranes, it cross-links membrane proteins efficiently (20). Although bands representing oligomeric forms of GAAP were detected in non-reducing gels without cross-linking, they were more prominent after incubation with DFDNB (Fig. 1*D*). A semi-logarithmic plot of the predicted number of cross-linked monomers in each band against its relative gel mobility was linear (Fig. 1*E*), consistent with the assigned subunit stoichiometry. The main bands detected were of ∼20–23, ∼37–45, ∼60, and ∼80 kDa, consistent with the presence of monomeric, dimeric, trimeric and tetrameric forms of GAAP.

The results using different methods applied to cell lysates and purified proteins demonstrate that GAAP forms tetramers. It is unclear whether the smaller oligomers and monomers are native states or result from incomplete retention of the tetramer during sample preparation, cross-linking, and gel analyses.

**FIGURE 2.**
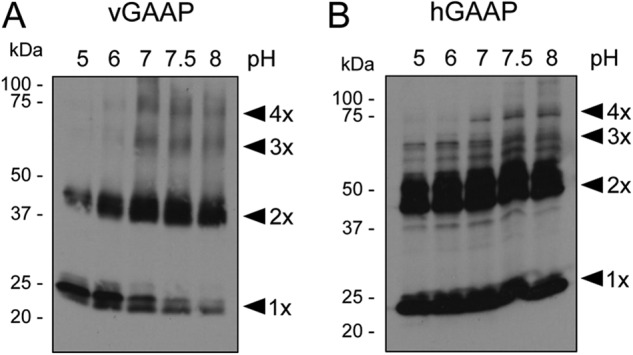
**Oligomerization of GAAP is pH-dependent.** HeLa cells were transfected with plasmids encoding vGAAP-HA (*A*) or hGAAP-HA (*B*), and after 18 h, cell lysates were prepared and incubated at varying pH, prior to SDS-PAGE under non-reducing conditions. Membranes were then immunoblotted with an anti-HA antibody. Results shown are representative of three independent experiments. *Arrowheads* show the different oligomeric states of GAAP. The sizes of molecular mass markers are shown in kDa.

The mechanism by which BI-1 allows Ca^2+^ to leak from the ER is pH-dependent. This was suggested to be directly related to increased oligomerization of BI-1 at lower pH (21). To investigate whether oligomerization of GAAPs was affected by pH, hGAAP-HA and vGAAP-HA were expressed in HeLa cells, extracted from membranes using CHAPS-based buffers of varying pH, and analyzed by non-reducing SDS-PAGE. Oligomerization of both vGAAP and hGAAP occurred within the physiological pH range (Fig. 2, *A* and *B*). At pH 6, vGAAP was mostly monomeric/dimeric, but higher oligomers formed as the pH increased. Oligomerization of vGAAP peaked at pH 7, whereas oligomerization of hGAAP increased up to pH 8.

##### Determinants of Oligomerization and Their Sensitivity to Reducing Agents Differ for vGAAP and hGAAP

**FIGURE 3.**
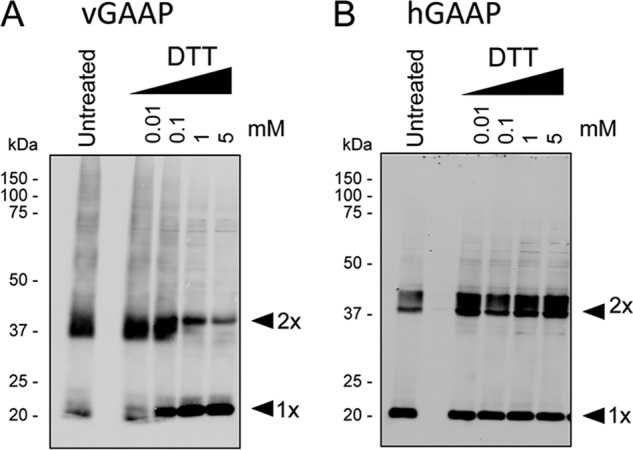
**DTT disrupts dimerization of vGAAP but not hGAAP.** HeLa cells were transfected with plasmids encoding vGAAP-HA (*A*) or hGAAP-HA (*B*). Cell lysates were prepared 18 h later and treated with DTT before analysis by SDS-PAGE and immunoblotting with anti-HA antibody. Results shown are representative of three independent experiments. *Arrowheads* show the different oligomeric states of GAAP. The sizes of molecular mass markers are shown in kDa.

Analysis of vGAAP and hGAAP by non-reducing SDS-PAGE identified monomeric and dimeric forms of GAAPs (Fig. 3, *A* and *B*, untreated lanes). Pretreatment of vGAAP with the reducing agent DTT caused a concentration-dependent loss of the band corresponding to dimeric GAAP and a concomitant increase in the monomeric band (Fig. 3*A*). DTT had no effect on the oligomerization of hGAAP (Fig. 3*B*). These results suggest that dimerization of vGAAP, but not of hGAAP, may require disulfide bonds between cysteines.

**FIGURE 4.**
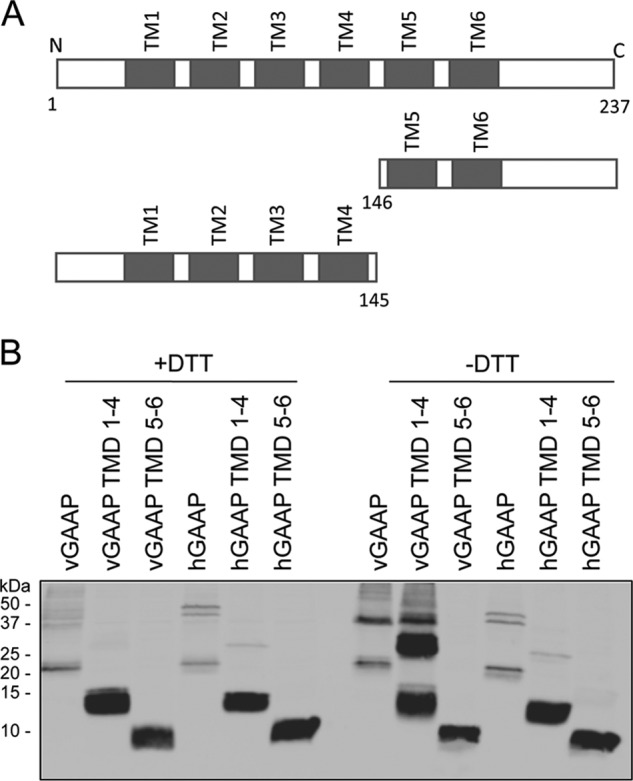
**The N-terminal and TMD1–4 of vGAAP are sufficient for dimerization.** *A*, wild type and truncated forms of vGAAP and hGAAP. *B*, lysates were prepared from HeLa cells 18 h after transfection with plasmids encoding wild type or truncated forms of vGAAP-HA and hGAAP-HA and were analyzed by SDS-PAGE under reducing (+DTT) or non-reducing (−DTT) conditions. Membranes were blotted with an anti-HA antibody to detect GAAP. The sizes of molecular mass markers are shown in kDa. Results shown are representative of three independent experiments.

Truncated forms of vGAAP and hGAAP containing either the N terminus and TMD1–4 (termed TMD1–4) or TMD5–6 and the C terminus (termed TMD5–6) (Fig. 4*A*) were used to investigate the domains required for oligomerization. These proteins were expressed in HeLa cells, and lysates were analyzed by reducing and non-reducing SDS-PAGE. vGAAP TMD1–4 formed oligomers, notably dimers, that were not detected in the presence of the reducing agent, DTT (Fig. 4*B*). In contrast, vGAAP TMD5–6 did not form oligomers (Fig. 4*B*). Although hGAAP TMD1–4 retained some marginal ability to dimerize, hGAAP TMD5–6 did not form oligomers (Fig. 4*B*). The effects of the truncations on folding of hGAAP are unknown. These data suggest that the first 145 amino acid residues of vGAAP are sufficient for dimerization, whereas the last two TMDs and the C-terminal portion of vGAAP are not required.

##### Oligomerization of vGAAP, but Not of hGAAP, Requires Cysteines

The overall level of amino acid conservation between TMBIM proteins is extremely high. However, there is no obvious conservation of cysteine residues between members of the GAAP subfamily (supplemental Fig. S1) or between members of the TMBIM family (supplemental Fig. S2), but vGAAPs have more cysteines than other GAAP family members (supplemental Fig. S1). Most of the unique cysteines in vGAAP are located near its N terminus, and based on the topology of GAAPs (9), only two of these (Cys^9^ and Cys^60^) are not embedded in the predicted TMDs (see Fig. 6*A*).

**FIGURE 5.**
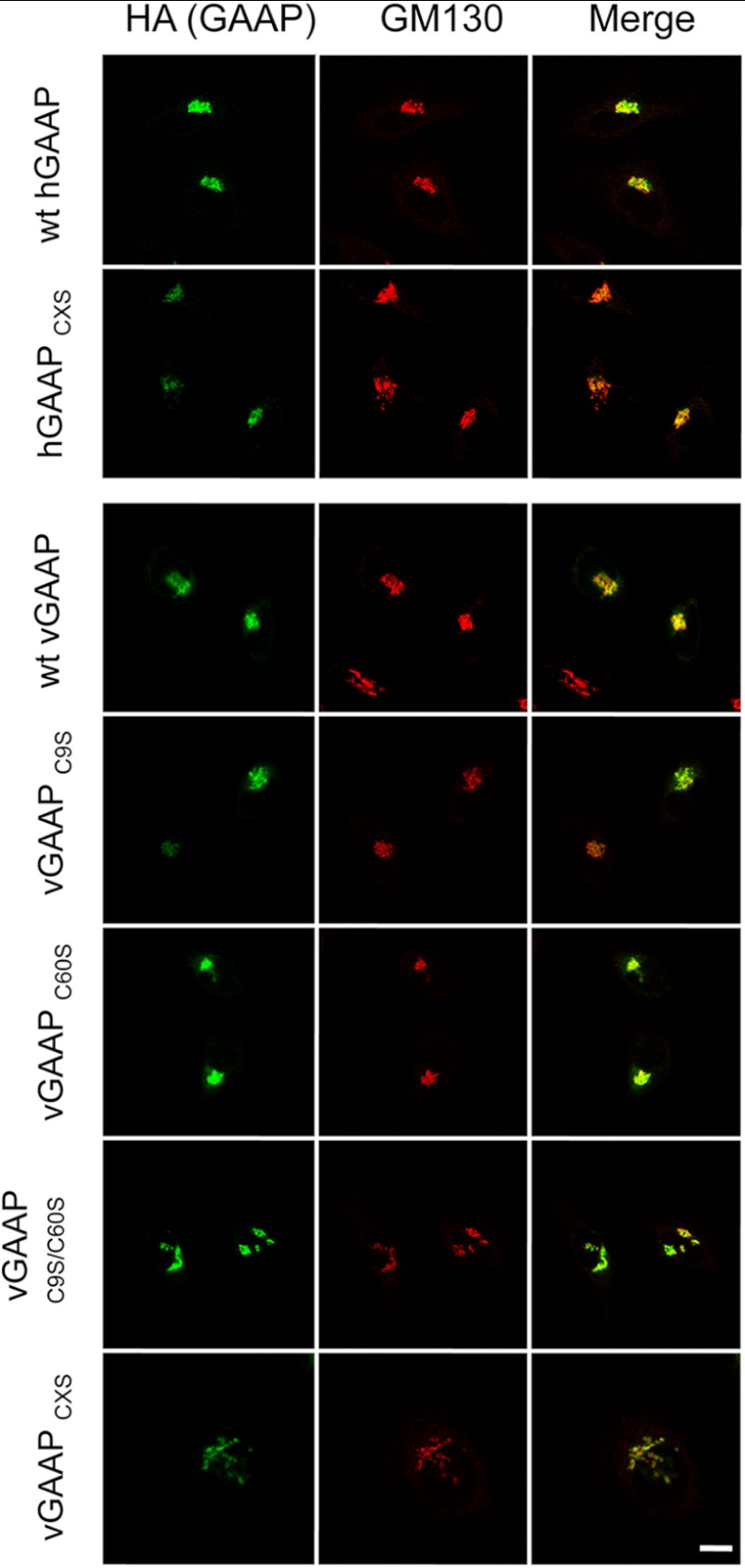
**Mutant hGAAP and vGAAP are expressed in the Golgi.** HeLa cells were fixed 16 h after transfection with plasmids encoding wild type or mutant vGAAP-HA or hGAAP-HA. Anti-HA antibody was used to detect GAAP, and anti-GM130 antibody was used to identify the Golgi. Cells were imaged using confocal microscopy. CXS denotes mutants with all cysteines mutated to serines. *Scale bar*, 10 μm.

**FIGURE 6.**
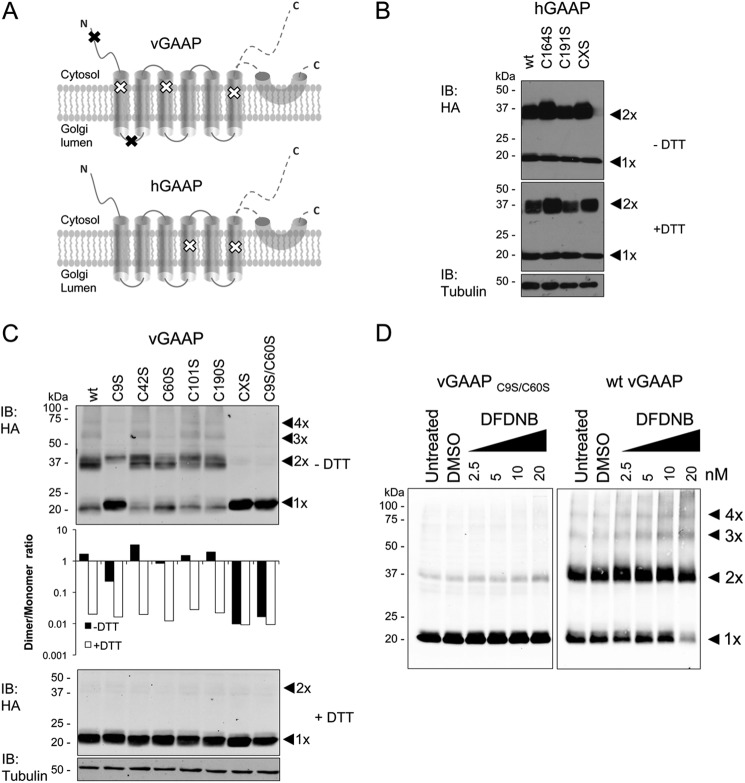
**Oligomerization of vGAAP requires cysteine residues 9 and 60.** *A*, distribution of cysteine residues in vGAAP and hGAAP. *Black* and *white x symbols* represent cysteine residues that are (*black*) or are not (*white*) needed for oligomerization of vGAAP. GAAP topology was described previously (9), where the region after TMD6 was suggested to be either a re-entrant loop or cytosolic. Cysteine to serine mutations were made in hGAAP-HA (*B*) or vGAAP-HA (*C*). Lysates from HeLa cells transfected with wild type or mutant vGAAP-HA or hGAAP-HA were analyzed by SDS-PAGE with or without DTT and immunoblotted (*IB*) with anti-HA antibody. *C*, *top panel*: dimer and monomer band intensities of the vGAAP-HA mutants were measured using Li-COR and plotted as the ratio between dimer and monomer (*C*, *middle panel*). CXS denotes mutants with all cysteines mutated to serines. *D*, lysates from HeLa cells transfected with wild type or C9S/C60S vGAAP-HA were incubated with DFDNB, resolved by non-reducing SDS-PAGE, and immunoblotted with anti-HA antibody. The predicted oligomeric states of vGAAP are shown by *arrowheads*. Results shown are representative of three independent experiments. The sizes of molecular mass markers are shown in kDa. *DMSO*, dimethyl sulfoxide.

To investigate the role of disulfide bonds in vGAAP and hGAAP oligomerization, we expressed GAAPs in which all cysteines were mutated to serines (CXS proteins), and GAAPs in which each cysteine was mutated individually to serine. Immunofluorescence confirmed that all mutant proteins were expressed in the Golgi (Fig. 5), suggesting normal folding and intracellular transport. To investigate the contribution of cysteine residues to dimerization of hGAAP and vGAAP, each mutant was expressed in HeLa cells and analyzed by reducing and non-reducing SDS-PAGE (Fig. 6, *B* and *C*). None of the hGAAP cysteine mutants, including hGAAP CXS, lost the ability to form dimers (Fig. 6*B*). On the contrary, loss of cysteines 9 or 60 from vGAAP reduced the dimer-to-monomer band ratio detected under non-reducing conditions (Fig. 6*C*). The vGAAP mutants C9S, C60S, C9S/C60S, and CXS also reduced the intensity of the trimer and tetramer bands (Fig. 6*C*). Simultaneous mutation of both critical cysteine residues in vGAAP (C9S/C60S) mimicked the CXS form and abolished dimerization (Fig. 6*C*). Addition of DTT showed that the expression levels of wild type and mutant vGAAPs were similar (Fig. 6*C*). The vGAAP C9S/C60S mutations also prevented appearance of dimeric, trimeric, and tetrameric cross-linked proteins after treatment with DFDNB (Fig. 6*D*). This demonstrates that Cys^9^ and Cys^60^ are the only cysteines involved in dimerization of vGAAP.

##### vGAAP Oligomerizes in Native Golgi Membranes

**FIGURE 7.**
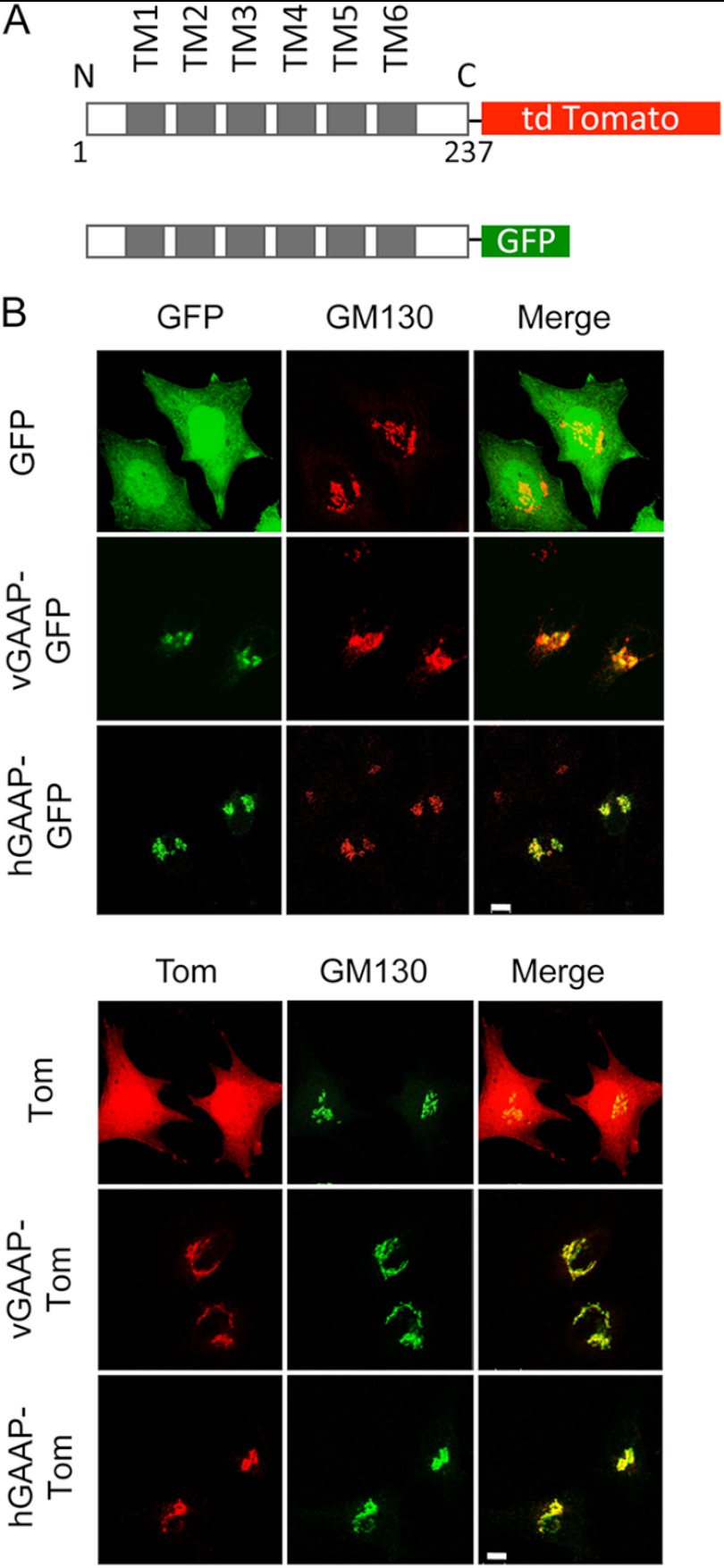
**GAAP-GFP and GAAP-Tom fusion proteins are expressed in the Golgi.** *A*, schematic representation of the GFP- and Tom-tagged GAAPs used for FRET analyses. *B*, HeLa cells were transfected with plasmids encoding GFP- or Tom-tagged vGAAP or hGAAP or with GFP or Tom alone, and 16 h after transfection, they were fixed and imaged by confocal microscopy. Golgi localization of the GAAP-GFP and the GAAP-Tom fusion proteins was assessed by comparison with immunostaining with anti-GM130 antibody. *Scale bars*, 10 μm.

**FIGURE 8.**
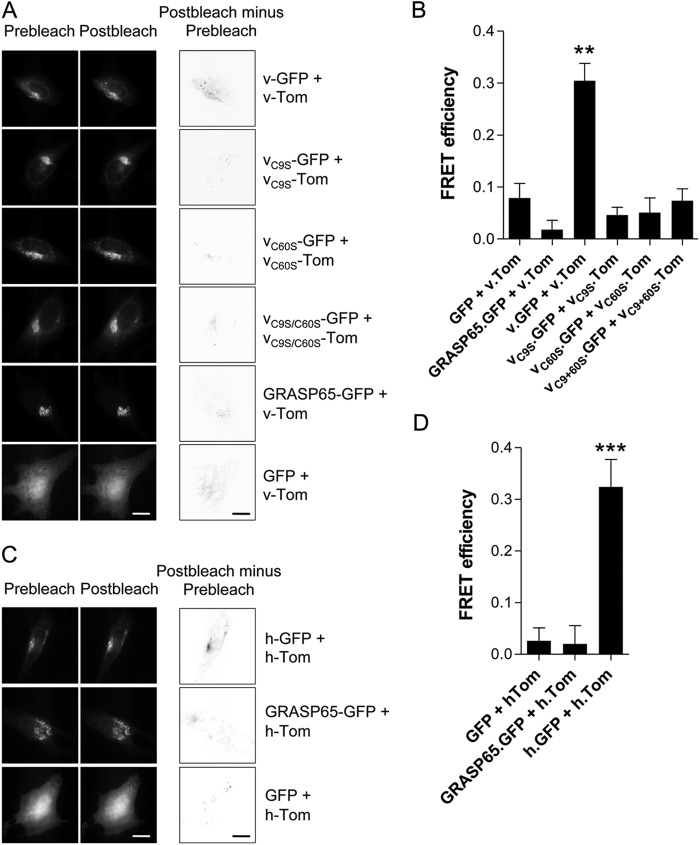
**GAAP interactions within native Golgi membranes.** FRET was detected by the increase in GFP fluorescence after photobleaching of Tom in HeLa cells transfected with plasmids encoding vGAAP-GFP or hGAAP-GFP with vGAAP-Tom or hGAAP-Tom or with control tagged proteins. Cells were fixed and imaged by wide field microscopy 16 h after transfection. GRASP65-GFP and GFP are negative controls. *A*, typical images of GFP fluorescence before and after photobleaching of Tom are shown for the indicated pairs of proteins. *Gray* value images (*third column*) were obtained by pixel-by-pixel subtraction of the GFP fluorescence before photobleaching from that measured after bleaching of Tom. *Scale bars*, 10 μm. *B*, summary results show FRET efficiency determined from subcellular regions of at least 10 cells where GFP and Tom colocalized. *C* and *D*, similar analyses for hGAAP show typical images (*C*) and analysis of FRET efficiency (*D*). Results shown are representative of three independent experiments. Results (*B* and *D*) show means ± S.E. from at least 10 cells and were analyzed using an unpaired Student's *t* test, with statistical significance shown as **, *p* < 0.01 or ***, *p* < 0.001 relative to GRASP65-GFP with vGAAP-Tom or hGAAP-Tom.

To analyze oligomerization of GAAP in native Golgi membranes, hGAAP, vGAAP, vGAAP C9S, vGAAP C60S, and vGAAP C9S/C60S were expressed with C-terminal tags of GFP or Tom for FRET analysis (Fig. 7*A*) (17). Immunofluorescence confirmed that each fusion protein co-localized with GM130, a marker of the Golgi apparatus (Fig. 7*B*) and so were transported normally. When vGAAP-GFP was co-transfected with vGAAP-Tom, a FRET signal was detected as an increase in GFP fluorescence after photobleaching of Tom. This was detected as both an increase in total cell vGAAP-GFP fluorescence after photobleaching of vGAAP-Tom (Fig. 8*A*) or by calculation of FRET efficiency from regions of the Golgi apparatus that expressed both fluorophores (Fig. 8*B*). The FRET signal between GFP- and Tom-tagged vGAAP was significantly greater than that between vGAAP-Tom and the control proteins, GFP or GRASP65-GFP (a Golgi membrane protein) (Fig. 8*B*) (22). All vGAAP-GFP cysteine mutants tested produced a FRET signal similar to the negative control pair of vGAAP-Tom with GRASP65-GFP (Fig. 8, *A* and *B*). These results demonstrate that within native Golgi membranes vGAAP and hGAAP form homologous associations (Fig. 8, *C* and *D*) and that for vGAAP cysteines Cys^9^ and Cys^60^ are required for oligomerization (Fig. 8, *A* and *B*). These results are consistent with those obtained using cross-linking and non-denaturing gels (Fig. 6, *C* and *D*). Both sets of results indicate that vGAAP and hGAAP form homo-oligomeric complexes, although the requirements for oligomerization are different.

##### Monomeric vGAAP Is Anti-Apoptotic and Depletes Intracellular Ca^2+^ Stores

The results so far demonstrate that GAAPs form homo-oligomers *in vitro* and in cell Golgi membranes, but they do not establish whether oligomeric and monomeric GAAPs co-exist in native membranes or which form is functional. It was, therefore, essential to show whether monomeric GAAP is functional and mimics native GAAP in both inhibiting apoptosis and depleting intracellular stores of Ca^2+^. Our results with cysteine mutants of vGAAP provide the first opportunity to address this because vGAAP C9S/C60S is appropriately expressed in the Golgi apparatus, but only as a monomer (Figs. 5 and 6).

**FIGURE 9.**
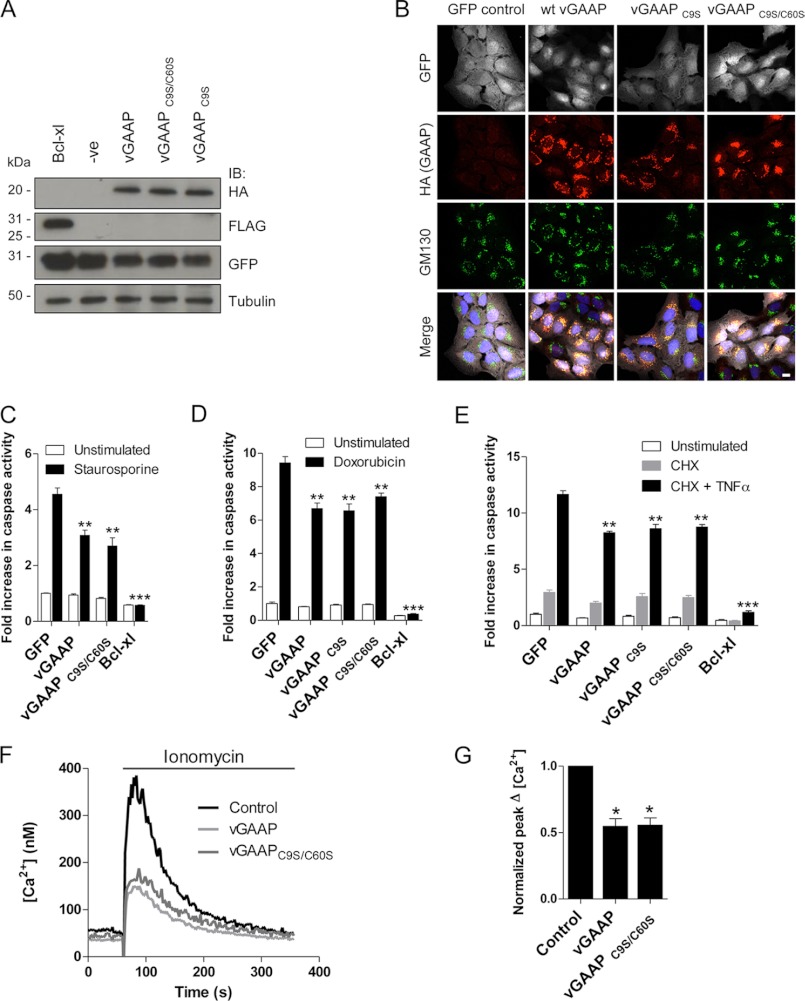
**Monomeric vGAAP protects cells from apoptosis and decreases the Ca^2+^ content of intracellular stores.** U2-OS cells were transduced with a bicistronic lentivirus encoding GFP only, or GFP with vGAAP-HA, vGAAP-HA C9S, vGAAP-HA C9S/C60S, or Bcl-x_L_-FLAG. *A*, after cell sorting to isolate GFP-expressing cells, lysates were immunoblotted (*IB*) with anti-GFP, anti-HA, and anti-FLAG antibodies. The sizes of molecular mass markers are shown in kDa. *B*, anti-HA antibody was used to detect GAAP and anti-GM130 antibody was used as a Golgi marker in cells prior to cell sorting. Cells were imaged using confocal microscopy. *Scale bar*, 10 μm. *C–E*, U2-OS cells stably expressing wild type or mutant vGAAPs were stimulated with staurosporine (0.5 μm, 6 h; *C*), doxorubicin (3 μm, 48 h; *D*), or TNFα (10 ng/ml) with cycloheximide (*CHX*) (20 μg/ml) for 16 h (*E*), and caspase activity was monitored. Results shown are representative of three independent experiments. *F* and *G*, typical increases in [Ca^2+^]*_i_* evoked by addition of ionomycin (1 μm) to populations of transfected HeLa cells in Ca^2+^-free medium (*F*). Summary results show peak increases in [Ca^2+^]*_i_* normalized to parallel measurements from mock-transfected cells. Results show means ± S.E. from five independent experiments. *Asterisks* (*C*, *D*, and *G*) indicate values that are significantly different from control: *, *p* < 0.05; **, *p* < 0.01; ***, *p* < 0.001.

The anti-apoptotic activity of GAAPs was described initially in human osteosarcoma U2-OS cells (1). To investigate the anti-apoptotic activity of wild type and mutant vGAAPs, U2-OS cells were transduced with lentivirus co-expressing GFP with either wild type vGAAP or vGAAP C9S/C60S and then sorted to select GFP-expressing cells. Lentiviruses expressing only GFP or Bcl-x_L_ were included as controls. Immunoblotting and immunofluorescence confirmed that vGAAP and vGAAP C9S/C60S were expressed at similar levels (Fig. 9*A*) and in the Golgi apparatus (Fig. 9*B*). Transduced U2-OS cells were treated with staurosporine, and caspase-3 and -7 activity was measured. As expected Bcl-x_L_, and to a lesser extent wild type vGAAP, reduced staurosporine-induced apoptosis (Fig. 9*C*). Inhibition of apoptosis by vGAAP C9S and vGAAP C9S/C60S was indistinguishable from wild type vGAAP, indicating that monomeric vGAAP is anti-apoptotic. Similar results were obtained when doxorubicin was used to stimulate the intrinsic apoptotic pathway (Fig. 9*D*). Furthermore, when cell death was triggered extrinsically using cycloheximide and TNFα, both wild type, vGAAP C9S, and vGAAP C9S/C60S provided the same protection from caspase activation (Fig. 9*E*).

To determine whether monomeric vGAAP regulates intracellular Ca^2+^ stores, HeLa cells were transfected with wild type vGAAP or vGAAP C9S/C60S, and the Ca^2+^ content of the intracellular stores was assessed by addition of ionomycin to cells in Ca^2+^-free medium (Fig. 9*F*). Both wild type vGAAP and vGAAP C9S/C60S significantly reduced the peak Ca^2+^ signals evoked by ionomycin (Fig. 9, *F* and *G*). Collectively, these results demonstrate that monomeric vGAAP inhibits apoptosis and reduces the Ca^2+^ content of the intracellular stores.

## DISCUSSION

Using biochemical and cell-based approaches, vGAAP and hGAAP were shown to form oligomers *in vitro* (Figs. 1–6) and in cell membranes (Fig. 8). Tetrameric GAAP was the largest oligomeric form to be readily detected (Figs. 1 and 2), but dimers were most abundant, and monomers were also evident (Figs. 1–4 and 6). Further analyses are required to establish whether the relative abundance of dimers and tetramers detected in gels faithfully reflects their prevalence in cells or results from incomplete preservation of oligomers during analysis *in vitro*. It is, however, clear that GAAPs form homo-oligomers in cells.

Most TMBIM proteins show several bands in SDS-PAGE suggesting that oligomerization may be a conserved property (6, 21). However, the determinants of oligomerization differ for vGAAP and hGAAP. The N-terminal region of vGAAP formed oligomers, whereas the equivalent region of hGAAP did not (Fig. 4*B*). Furthermore, oligomerization of vGAAP, but not of hGAAP, required disulfide bonds (Fig. 6, *B* and *C*). Cysteines 9 and 60 of vGAAP were essential for oligomerization of vGAAP whether assessed *in vitro* (Fig. 6) or in cells (Fig. 8). Despite the high level of overall sequence identity between members of the TMBIM sub-family, these cysteine residues are poorly conserved between GAAPs (supplemental Fig. S1). The mechanism by which hGAAP forms oligomers is presently unclear but clearly independent of disulfide bridges (Figs. 3*B* and 4*B*). Although it is not possible to rule out a contribution from coiled-coil, hydrophobic, ionic, or other protein-protein interaction elements in GAAP oligomerization, the results obtained by FRET show that disruption of disulfide bridges is sufficient to prevent GAAP oligomerization in the Golgi membrane.

BI-1, another member of the TMBIM family with functional similarity to GAAP, also forms dimers and tetramers (23). The mechanisms are unresolved (5, 12, 21), but it has been proposed that the oligomeric forms mediate ion transport (12, 23). Although oligomerization of TMBIM family members appears to be a conserved feature, the mechanisms vary and there may also be different functional consequences for the different oligomeric states of TMBIM proteins.

Our identification of point mutations that prevent oligomerization of vGAAP (Figs. 6 and 8) allowed direct assessment of the activity of a monomeric GAAP. Surprisingly, monomeric vGAAP mimicked wild type vGAAP in both reducing the Ca^2+^ content of intracellular stores and in protecting cells from apoptosis (Fig. 9). There are precedents with other oligomeric proteins that retain their function as monomers (24–26). In some cases, oligomerization allows differential activity (27, 28) or trafficking (29, 30). For example, some ion exchangers can assemble into oligomeric structures that modulate ion flux, but the oligomers are not absolutely required for function (31, 32). Oligomerization of GAAPs may likewise confer additional modulation or undefined functions, without being required to protect cells from apoptosis. It is notable that different TMBIM family proteins, GRINA and BI-1, are proposed to form hetero-oligomers that allow further regulation of activity (6). For GAAPs, it is also conceivable that the different oligomeric forms may have additional, possibly regulatory, functions.

Oligomerization of GAAPs and BI-1 is pH-dependent but with contrasting pH-sensitivities. Low pH favors oligomerization of BI-1 (21), whereas oligomerization of GAAP is more pronounced at high pH (Fig. 2). It is not yet clear whether this pH regulation, the only regulatory mechanism so far identified for GAAP, is exercised at the cytosolic or luminal surface of GAAP. Resting cytosolic pH is typically 7.2, but it is higher in solid tumor cells (33), and it is dynamically regulated by extracellular stimuli in many cells (34). Golgi pH fluctuates between 6 and 6.7 (35). Across these pH ranges, a substantial fraction of GAAP is monomeric (Fig. 2), consistent with Golgi-localized GAAP monomers regulating cellular behavior. However, physiological changes in cytosolic or Golgi pH may redistribute GAAP between its active monomeric form and oligomers that may be inactive or behave differently. It was reported that BI-1 oligomerization peaks at pH 5, very different from the physiological pH of ER of ∼7 (35), at which BI-1 was shown to be monomeric (21). These discrepancies might suggest that the monomeric form of BI-1 could still be functional, as seen for GAAP.

In summary, this work has shown that oligomerization of GAAPs, conserved members of the TMBIM protein family, is regulated by physiological changes in pH, and has established the determinants of oligomerization. Mutation of residues required for oligomerization of vGAAP produced a monomeric vGAAP, and use of this form established that monomeric GAAP is functional in both protecting cells from apoptosis and in reducing the Ca^2+^ content of the ER. This is the first demonstration that a monomeric TMBIM family member is functional.

## Footnotes

4 The abbreviations used are:

GAAP: Golgi anti-apoptotic protein
BI-1: Bax inhibitor-1
[Ca^2+^]*_i_*: intracellular free Ca^2+^ concentration
CMLV: camelpox virus
DFDNB: 1,5-difluoro-2,4-dinitrobenzene
ER: endoplasmic reticulum
vGAAP: viral GAAP
hGAAP: human GAAP
TMBIM: transmembrane BI-1-containing motif
TMD: transmembrane domain
Tom: tandem tomato
VACV: vaccinia virus.
